# FKN/CX3CR1 axis facilitates migraine-Like behaviour by activating thalamic-cortical network microglia in status epilepticus model rats

**DOI:** 10.1186/s10194-022-01416-w

**Published:** 2022-04-05

**Authors:** Yanjie Zhou, Lily Zhang, Yuyan Hao, Liu Yang, Shanghua Fan, Zheman Xiao

**Affiliations:** Department of Neurology, Renmin Hospital of Wuhan University, Wuhan University, 99 Zhangzhidong Road, Wuhan, 430060 China

**Keywords:** Migraine, Epilepsy, Microglia, FKN, CX3CR1, BDNF

## Abstract

**Background:**

The incidence of migraines is higher among individuals with epilepsy than in healthy individuals, and these two diseases are thought to shared pathophysiological mechanisms. Excitation/inhibition imbalance plays an essential role in the comorbidity of epilepsy and migraine. Microglial activation is crucial for abnormal neuronal signal transmission. However, it remains unclear whether and how microglia are activated and their role in comorbidities after being activated. This study aimed to explore the characteristics and mechanism of microglial activation after seizures and their effect on migraine.

**Methods:**

Model rats of status epilepticus (SE) induced by intraperitoneal injection of lithium chloride (LiCl)-pilocarpine and migraine induced by repeated dural injections of inflammatory soup (IS) were generated, and molecular and histopathologic evidence of the microglial activation targets of fractalkine (FKN) signalling were examined. HT22-BV2 transwell coculture assays were used to explore the interaction between neurons and microglia. LPS (a microglial agonist) and FKN stimulation of BV2 microglial cells were used to evaluate changes in BDNF levels after microglial activation.

**Results:**

Microglia were specifically hyperplastic and activated in the temporal lobe cortex, thalamus, and spinal trigeminal nucleus caudalis (sp5c), accompanied by the upregulation of FKN and CX3CR1 four days after seizures. Moreover, SE-induced increases in nociceptive behaviour and FKN/CX3CR1 axis expression in migraine model rats. AZD8797 (a CX3CR1 inhibitor) prevented the worsening of hyperalgesia and microglial activation in migraine model rats after seizures, while FKN infusion in migraine model rats exacerbated hyperalgesia and microglial activation associated with BDNF-Trkb signalling. Furthermore, in neuron-microglia cocultures, microglial activation and FKN/CX3CR1/BDNF/iba1 expression were increased compared with those in microglial cultures alone. Activating microglia with LPS and FKN increased BDNF synthesis in BV2 microglia.

**Conclusions:**

Our results indicated that epilepsy facilitated migraine through FKN/CX3CR1 axis-mediated microglial activation in the cortex/thalamus/sp5c, which was accompanied by BDNF release. Blocking the FKN/CX3CR1 axis and microglial activation are potential therapeutic strategies for preventing and treating migraine in patients with epilepsy.

**Supplementary Information:**

The online version contains supplementary material available at 10.1186/s10194-022-01416-w.

## Introduction

Migraine and epilepsy are among the most frequently encountered nervous system diseases. A large number of studies have highlighted the complex relationship between migraine and epilepsy. Patients with epilepsy have a 52% greater lifetime prevalence of migraines than those without epilepsy [1]. The underlying mechanisms are linked to many factors, such as direct causality, common environmental risk factors, and common genetic susceptibility [2]. Current studies tend to explain this comorbidity in terms of the imbalance between neuronal excitation and inhibition [2, 3]. Abnormalities in neuronal excitability abnormalities exist in the cortex and thalamus in both epileptic and migraine patients, suggesting that the thalamic-cortical network may play a role in the comorbidity [4, 5].

Resting-state functional magnetic resonance imaging (fMRI) has revealed diverse connections between the thalamus and cortex in humans [6]. Epilepsy originates from abnormal dynamics in neuronal networks. A sudden discharge of action potentials during epileptic seizures increases neural synchronization and enhances the rhythmic network activity of the cortex and thalamus [7]. In migraine, cortical spreading depression (CSD), which is thought to be the neurophysiological correlate of migraine [8], may activate and sensitize trigeminovascular pain pathway by inducing a cortical neuroinflammatory cascade [9]. Numerous studies have suggested that trigeminal neurovascular system changes may lead to neuronal abnormalities in genetically susceptible individuals that are directly related to thalamic cortical rhythmicity [10]. Rhythmic cortical feedback to the thalamus is critical in amplifying thalamocortical activity, making it a strong candidate for influencing neuronal excitability [4].

Microglia and neuron-microglia interactions are crucial in the central nervous system (CNS) and are characterized by altered neural network excitability [11]. Many studies have indicated that microglial activation is associated with epileptogenesis and migraine attacks [12, 13]. Seizures can induce microglial activation in experimental models and human epilepsy [14, 15]. After a seizure, microglia remain morphologically activated for a long time, as if in a primed state [15]. In addition, recent studies have shown that microglial activation and neuroinflammation are critical factors in the generation of the pain associated with migraine [16]. Activated microglia can release proinflammatory molecules with neuromodulatory properties, thereby reducing seizure thresholds [17]. This finding suggests that microglia may be involved in the shared mechanisms between migraine and epilepsy. However, comparatively little attention has been given to the role of microglia in the common mechanisms of epilepsy and migraine. It would be interesting to explore the role of microglia in migraine after seizures.

FKN is a chemokine expressed by both neurons and glia, and its receptor, CX3CR1, is primarily expressed on microglia [18]. Given the distinct expression of FKN and its receptor in neurons and microglia, reducing the FKN/CX3CR1 axis is sufficient to affect certain physiological processes [19]. FKN acts as a regulator of microglial activation within the CNS [20]. Through the FKN/CX3CR1 axis, microglial communication with neuronal elements can monitor and alter synaptic activity under epileptic conditions [20]. Neuroimmune communication mediated by the FKN/CX3CR1 axis also leads to increased nociception in various forms of neuropathic pain [21]. However, the role of this axis in migraines and whether it is associated with increased susceptibility to migraines after the onset of epilepsy remain unknown.

Brain-derived neurotrophic factor (BDNF), including the precursor molecule (proBDNF) and a mature form, is mainly expressed in the CNS. BDNF is released by neurons and microglia and plays a crucial role in neuroinflammation [22]. Many studies have shown that BDNF participates in many nervous system diseases, including epilepsy and migraine [23]. Some studies indicate that activated microglia can release increased amounts of BDNF [24]. This finding suggests that microglial activation after epilepsy may facilitate the occurrence of migraines through BDNF signalling.

Our research aimed to investigate whether microglia and their interactions with neurons via the FKN/CX3CR1 axis in the thalamic cortical network are involved in the comorbidities of epilepsy and migraine by regulating the expression of BDNF. Here, we used a comorbidity model of epilepsy and migraine established in our previous research [25]. Migraine- and epilepsy- relevant behaviour and molecular biological alterations were investigated in the cortex/thalamus/sp5c.

## Materials and methods

### Animals

Adult male Sprague–Dawley (SD) rats weighing 200–250 g were procured from the Laboratory Animal Centre of Renmin Hospital, Wuhan University. All rats were housed under specific pathogen-free (SPF) laboratory conditions with a 12-h light/dark cycle (lights on at 8:00 A.M., humidity 60 ± 5%, temperature 22 ± 2 °C) and ad libitum access to food and water. The protocol was reviewed and approved by the Animal Care and Use Committee of Renmin Hospital of Wuhan University. Animal studies were performed in compliance with the ARRIVE guidelines and the Basel Declaration, including the “3R” concept [26].

### Status epilepticus induction and seizure quantification

The induction of status epilepticus using pilocarpine was performed as previously described [27]. Experimental rats were injected with lithium chloride (127 mg/kg, i.p., Sigma–Aldrich, St. Louis, MO), and 18 to 24 h later, atropine sulfate (1 mg/kg, i.p., Double-Crane pharmaceutical Co., Ltd) was administered to antagonize the peripheral cholinergic effect. Thirty minutes later, SE was induced by injecting pilocarpine hydrochloride (35 mg/kg, i.p., Sigma). Seizure behaviour was scored by the modified Racine scale with the following stages: (0) no abnormality, (1) mouth and facial movements, (2) head nodding, (3) forelimb clonus, (4) rearing, and (5) rearing and falling [28]. Pilocarpine hydrochloride was administered (10 mg/kg, i.p.) every 30 min until the rats developed seizures at Stage 4 of the Racine scale. Only rats that progressed to Stage 4 or greater were selected. The maximum dose for pilocarpine was 60 mg/kg. Diazepam (10 mg/kg, i.p., Sinopharm) was used to terminate seizures after a one-hour status epilepticus. Sham rats received the same treatment with lithium chloride and atropine sulfate, but an equivalent amount of phosphate buffered saline (PBS) was used instead of pilocarpine.

### Dural injection of inflammatory soup and nociceptive behavioural tests

The experimental procedures were carried out as previously reported [25]. After the skull was exposed, a 1 mm hole in the middle of the superior sagittal sinus (between bregma and lambda) was made with a precooled dental drill (DH-0 Pin Vise, Plastics One) to carefully expose the dura. A 0.5 mm guide cannula (22 GA, #C313G, Plastics One) was inserted into the hole and sealed into place with a mixture of dental cement powder and superglue. A dummy cannula (#C313DC, Plastics One Inc.) was inserted to ensure the patency of the guide cannula. The skin incision was closed with a silk suture. After one day of recovery from the surgery, 30 μl of IS (2 mM bradykinin, 2 mM serotonin, 2 mM histamine, and 0.2 mM prostaglandin E2) was administered via the annular tubes to stimulate the dura at a rate of 4 μl/minute once per day for four days according to previously described methods [16, 29]. Sham-operated rats underwent the same surgical procedure, but an equal amount of PBS was used instead of IS.

Nociceptive behaviour was assessed by counting the number of head-scratching actions and measuring the periorbital region mechanical sensitivity threshold. The head-scratching counts were recorded for one hour immediately after the last IS administration to assess nociceptive behaviours. Only rats scratching the face above the eye, which is innervated by the first division of the trigeminal nerve, were counted [30]. To assess the acute pain threshold, mechanical thresholds were measured by Von-Frey filaments after the last IS dural infusion. Testing began with the 1-gram filament on the face. If the rat showed a head-back reaction, the Von-Frey filament was changed to a smaller wire. If there was no reaction, the wire size was increased until the rat quickly retracted its head [31]. The pain threshold is determined by the first change in response. The evaluators were blinded to all experimental conditions.

### Drug administration

AZD8797 (an antagonist of CX3CR1) administration was carried out as described previously [32]. AZD8797 (HY-13848, MCE, USA) was dissolved in a DMSO solution to yield a final concentration of 2 mg/ml according to the instructions. Rats received either an intraperitoneal injection of AZD8797 (1 mg/kg) or an equal amount of PBS once per day for four days before dural IS administration. Rat fractalkine (PeproTech, USA) was dissolved in 0.9% NaCl to yield a final concentration of 1 μg/μl; bilateral intracerebral injection of fractalkine was performed stereotactically to the sp5c at the following coordinates: anteroposterior, − 14.08 mm; lateromedial, ± 2.75 mm; dorsoventral, − 8.65 mm relative to the bregma in the rat. A total of 2.5 μl was injected into each site using a 5 μl glass syringe with a fixed needle [33]. Rats in the sham groups received 2.5 μl of PBS at each site. A schematic representation of the intracerebral injection is given in Supplementary Fig. 1.

### Experimental design and animal groupings

After one week of acclimatization, a total of 80 rats were randomly divided into ten groups of eight rats each according to experimental needs.

#### Experiment 1

**Fig. 1:**
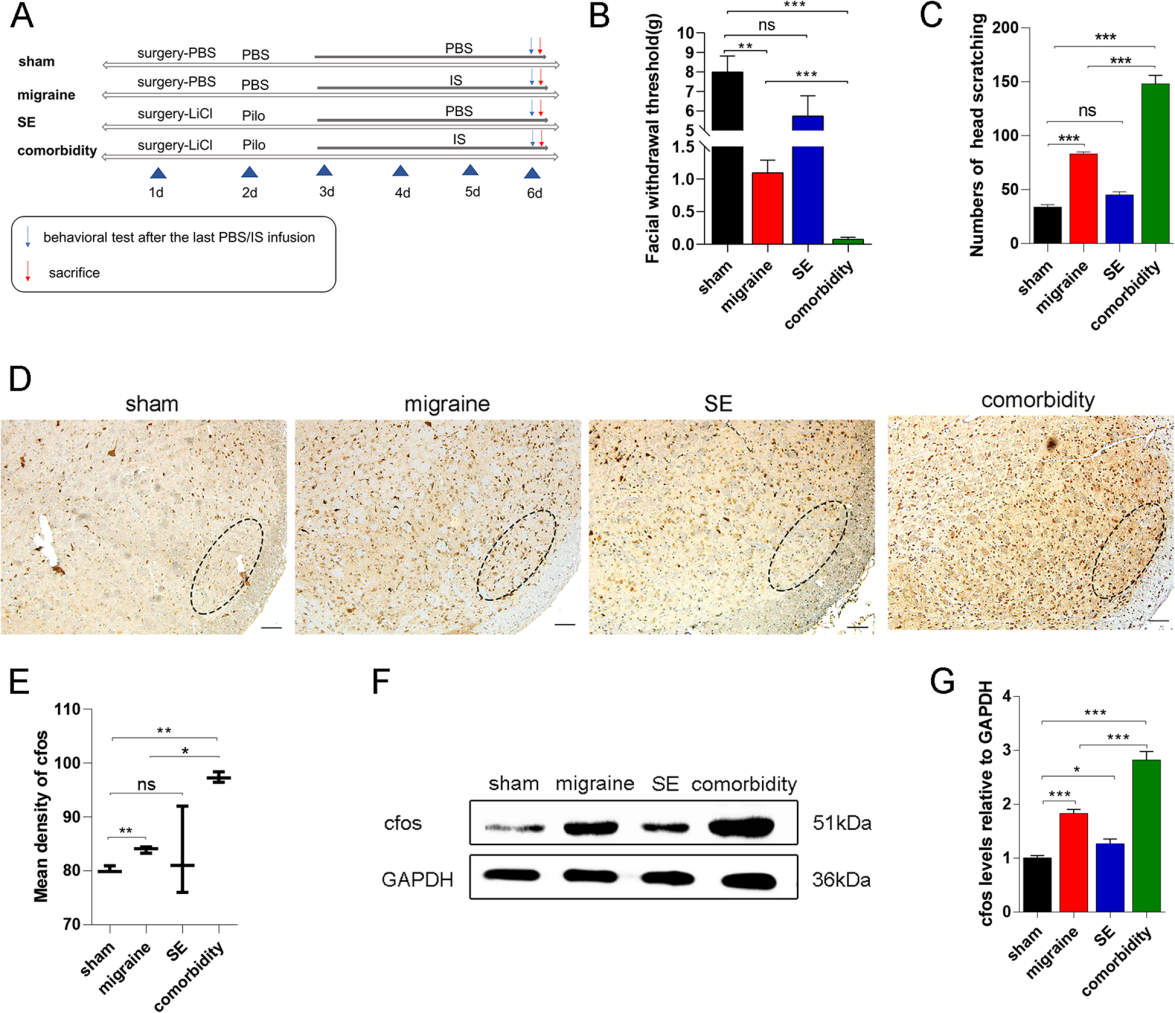
SE-induced increase in nociceptive behaviour in migraine model rats. **A** Flow diagram of the experimental design. **B, C** Facial mechanical threshold and head-scratching counts were assessed in the different groups. Mean ± SEM; one-way ANOVA, *n* = 6 rats per group; **P < 0.01, ***P < 0.001. **D** Representative immunohistochemical staining of cfos in the sp5c. Scale bars = 100 μm. **E** Quantification of cfos density in each group. Mean ± SEM; one-way ANOVA, *n* = 3 rats per group; *P < 0.05, **P < 0.01. **F, G** Representative western blot results for cfos in the different groups. Mean ± SEM; one-way ANOVA, *n* = 3 rats per group; *P < 0.05, ***P < 0.001

Experiment 1 was designed to assess the effect of seizures on migraine and the expression of the FKN/CX3CR1 axis in SE and migraine rats. The rats were randomly divided into four groups: sham group, migraine group, SE group, and comorbidity group. The sham group received PBS. Rats in the migraine group underwent IS infusion. LiCl-Pilo was used in the SE group. Repeated IS injection into the dura mater of SE model rats was performed once per day for four consecutive days to establish the comorbidity group (Fig. 1A). After evaluating the behaviour of each group, 3 ~ 4 rats (randomly selected) from each group were immediately used for perform western blotting (WB), and other rats were perfused and used to make paraffin sections for immunohistochemistry (IHC) or immunofluorescence (IF) analysis. In addition, the colocalization of CX3CR1 with microglia and FKN with neurons was evaluated using double immunofluorescence staining in the sham group, migraine group, and comorbidity group.

#### Experiment 2

Experiment 2 was performed to investigate whether the effect of seizures on migraine was mediated by FKN/CX3CR1. AZD8797 was administered to rats in the comorbidity group. The groups included the sham-PBS group, the comorbidity-PBS group, and the comorbidity-AZD group (Fig. 5A). After evaluating the behaviour of each group, we collected sp5c tissues for WB to examine the antagonistic effect of AZD8797 on FKN/CX3CR1 (*n* = 3 ~ 4/group). The protein levels of proBDNF/BDNF were also measured to examine the potential molecular pathway associated with the FKN/CX3CR1 axis. Moreover, IF staining was performed to examine changes in microglia (*n* = 3 ~ 4/group).

#### Experiment 3

To further validate the effect of FKN/CX3CR1 on facilitating migraine-related behaviours and the specific mechanisms, FKN was infused into rats with migraine. There were three groups: the PBS-sham group, the PBS-migraine group, and the FKN-migraine group (Fig. 6A). Behavioural assessment, WB (*n* = 3 ~ 4/group), and IF staining (*n* = 3 ~ 4/group) were conducted in this experiment.

The observers were blinded to group allocations during the animal experiments.

### Cell cultures and drug treatments

The BV2 mouse microglial cell line ICLCATL03001 (kindly provided by the Emergency Department Laboratory, Renmin Hospital of Wuhan University) was cultured in DMEM supplemented with 10% FBS, 100 U/ml penicillin, and 100 μg/ml streptomycin in a 5% CO_2_ incubator at 37 °C. BV2 cells were activated with lipopolysaccharide (LPS, 1 μg/ml, MCE, USA) for 90 min [34]. To examine the effects of FKN on microglia and the role of microglial release and synthesis of BDNF, we administered 0.1 mg/ml FKN (PeproTech, USA) to the cell cultures for 90 min according to the morphological changes in BV2 cells.

HT22 mouse hippocampal neurons were purchased from Wuhan Procell Century Technology. The cells were cultured in DMEM, supplemented with 10% FBS, 100 U/ml penicillin, and 100 U/ml streptomycin at 37 °C in a humidified environment containing 5% CO_2_.

### Neuron-BV2 microglia transwell coculture

Neuron-BV2 microglial transwell cultures were used to investigate the effect of neuron-mediated FKN on microglia. BV2 microglial cells were plated in 6-well dishes (10^6^ cells/well). HT22 mouse hippocampal neurons were plated (5 × 10^5^ cells/well) in transwell chambers (0.4 µm, Cell Biolabs, Inc.), and these inserts were placed on top of the wells containing microglia. After 24 h of coculture, morphological changes in microglia were observed. Microglia on the lower membrane were collected, and the related protein levels were measured.

### Western blotting

Rat brain tissue or cell samples were lysed in RIPA buffer containing phenylmethylsulfonyl fluoride (PMSF) and a protease inhibitor cocktail as previously described. Lysates were centrifuged and collected. Equal amounts of total protein ranging from 15 to 30 μg total protein were separated by SDS-PAGE, and blotted onto a polyvinylidene difluoride (PVDF) membrane (Millipore). The following antibodies were used: rabbit anti-cfos (1:1000, Abcam), rabbit anti-CX3CR1 (1:3000, Abcam), goat anti-FKN (1:200, Abcam), rabbit anti-BDNF (1:3000, Abcam), rabbit anti-GAPDH (1:3000, Servicebio), and goat anti-iba1 (1:500, Woko). Blots were developed with an HRP-labelled goat anti-rabbit antibody (1:5000, Servicebio) and an HRP-labelled donkey anti-goat secondary antibody (1:5000, Servicebio). Protein bands were visualized using a chemiluminescence system (ChemiDocTM XRS + , BioRad). Protein expression was semiquantitatively analysed with Image J software.

### Immunostaining

Paraffin-embedded serial sections and cells were fixed with paraformaldehyde and treated with 3% H_2_O_2_ for 10 min. Then, the sections were washed 3 times in PBS solution and blocked in 3% bovine serum albumin (BSA) and 0.3% Triton X-100 for 1 h. The sections were incubated overnight with rabbit anti-cfos (1:500, Abcam), rabbit anti-CX3CR1 (1:500, Abcam), goat anti-FKN (1:100, R&D), goat anti-iba1 (1:500, Woko), mouse anti-Neun (1:200, Abcam), and rabbit anti-BDNF (1:400, Abcam) at 4 ℃. For IHC, after being incubated with HRP-labelled goat anti-rabbit secondary antibodies (1:200, Servicebio) and HRP-labelled donkey anti-goat secondary antibodies (1: 200, Servicebio) for 1 h, the signal was developed according to the manufacturer's instructions for the High-Efficiency IHC Detection System Kit (Sharp, Wuhan China). For IF and immunocytochemistry (ICC) experiment, primary antibodies were detected with an appropriate fluorescent secondary antibody. Fluorescent secondary antibodies included FITC-labelled donkey anti-rabbit IgG (1:200, Servicebio), FITC- or Cy3-labelled donkey anti-goat IgG (1:200, Servicebio), Cy3-labeled donkey anti-mouse IgG (1:200, Servicebio). The slices were washed three times in PBS, covered with a glass cover with mounting solution and examined under a fluorescence microscope (Olympus BX51; Olympus, Tokyo, Japan).

### Sholl analysis

The complexity of microglia was analysed by setting a group of sequential concentric circles covering the cells with Image J software. The innermost circle (7 μm radius of activated microglia and 4 μm radius of resting microglia) was located in the nucleus. The other circles were arranged following a step length of 2 μm. The diameter of the outermost circles was adaptive according to each cell. Then, the number of intersections of individual concentric circles was counted.

### Statistical analysis

Statistical analyses were performed with SPSS version 22.0 and GraphPad Prism 8.0. Unpaired Student's t tests were used to compare the means of two groups. One-way ANOVA followed by Bonferroni post-hoc tests was used for comparisons among three or more groups. All data are presented as the mean ± SEM. The results were considered significant at *P < 0.05, **P < 0.01, and ***P < 0.001.

## Results

**Fig. 2:**
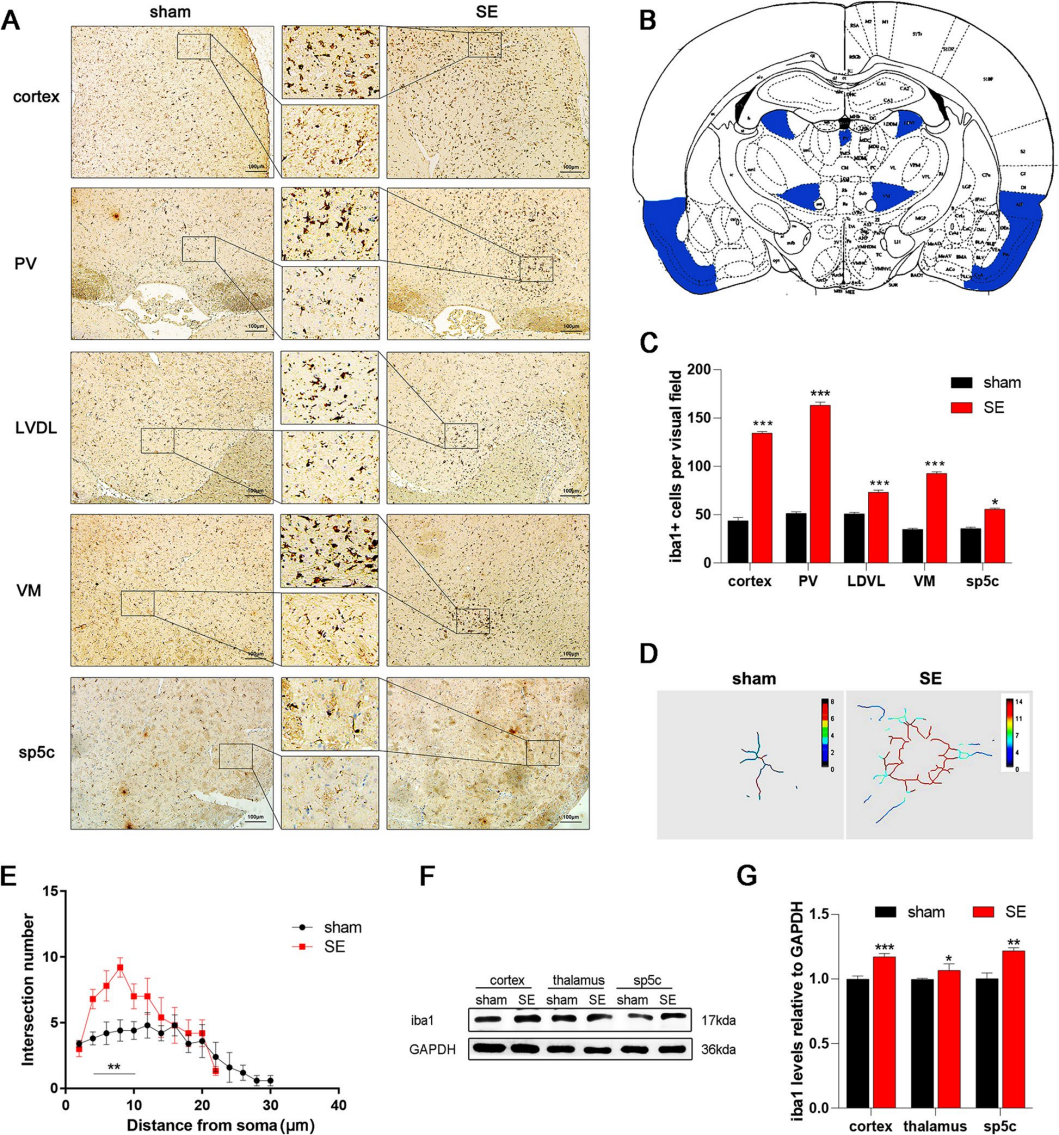
SE-induced increase in microglial activation and number in the cortex/thalamus/sp5c in adult rats. **A** Representative immunohistochemical staining of iba1 in the cortex, PV, LDVL, VM, and sp5c. Scale bars = 100 μm. **B** Iba1-positive (iba1 +) areas in different brain regions. **C** Quantification of the number of Iba1 + cells. Mean ± SEM; unpaired t test versus sham group; *n* = 3 rats per group. *P < 0.05, ***P < 0.001 compared to the sham group. **D-E** Sholl analysis of microglia showed that SE-induced branch shortening and increased synapses compared to those in the sham group. Mean ± SEM; unpaired t test versus sham group; *n* = 6–11 cells per group; **P < 0.01. **F** Western blot analysis of iba1 in the cortex, thalamus, and sp5c. **G** Western blot showing that the protein levels of iba1 in the cortex/-thalamus/sp5c were increased compared those in to the sham group. *n* = 3 rats per group. Mean ± SEM; unpaired t test versus sham group; *P < 0.05, **P < 0.01, ***P < 0.001 compared to the sham group

**Fig. 3:**
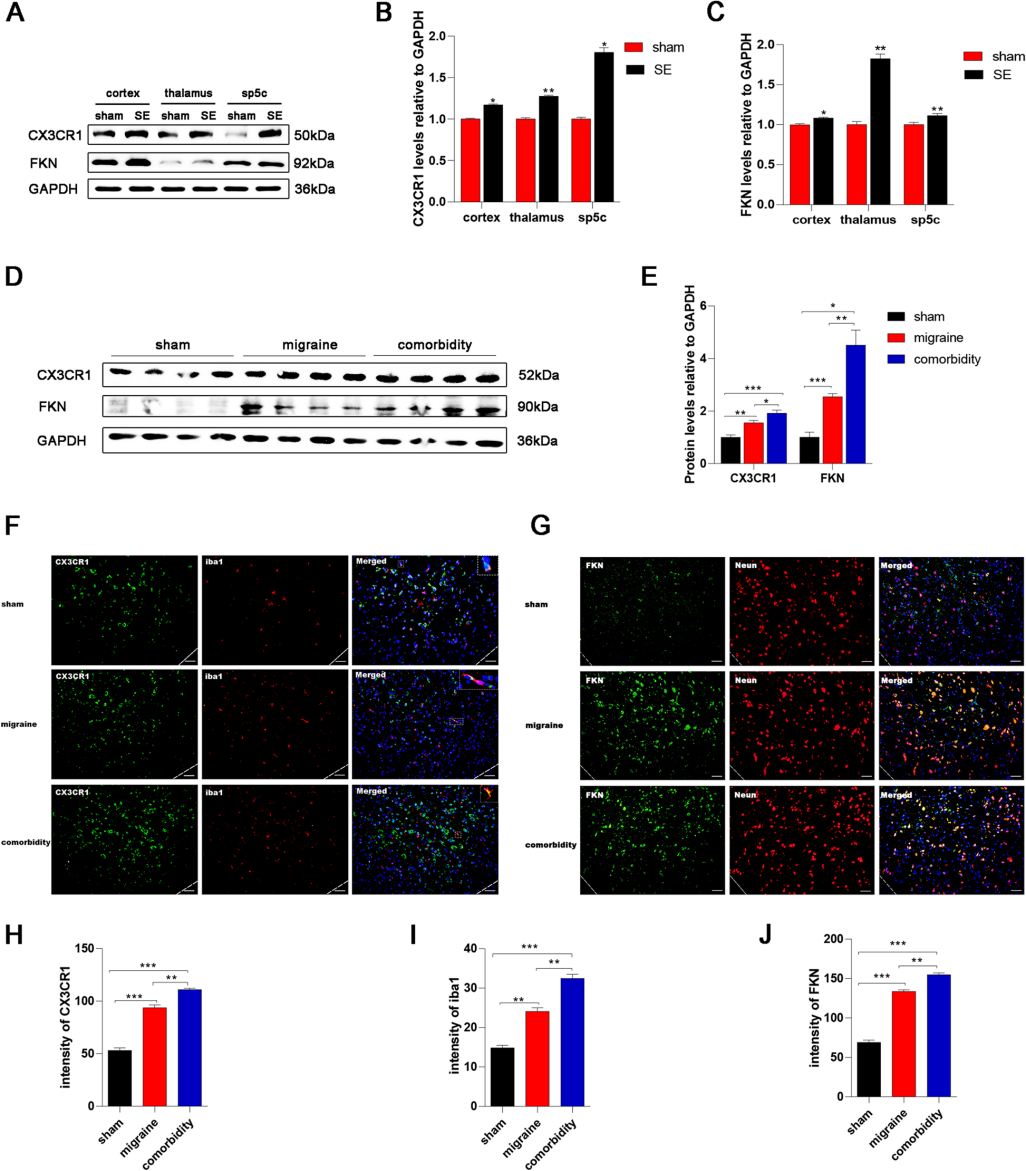
SE upregulates FKN/CX3CR1 in the sp5c of migraine model rats. **A** Representative western blot images showing CX3CR1 and FKN protein expression in the cortex/thalamus/sp5c of SE model rats. **B-C** Western blot analysis showing that CX3CR1 and FKN protein levels of in the cortex/thalamus/sp5c were upregulated in the SE group compared to the sham group. Mean ± SEM; unpaired t test versus sham group; *n* = 3 rats per group; *P < 0.05, **P < 0.01. **D** Representative western blots showing CX3CR1 and FKN in the different groups. **(E)** Western blot analysis showing that the protein levels of CX3CR1/FKN were increased in the migraine group, and further increased in the comorbidity group. Mean ± SEM; one-way ANOVA; *n* = 4 rats per group; *P < 0.05, **P < 0.01, ***P < 0.001. **F** Representative immunofluorescence images showing CX3CR1 + cells (green) colocalized with Iba1 + cells (red) in the sp5c of different groups. Scale bars = 50 μm. The white box was enlarged. **G** Representative immunofluorescence images showing FKN + cells (green) colocalized with Neun + cells (red) in the sp5c in the different groups. Scale bars = 50 μm. **H-I** Quantification of CX3CR1 and iba1 immunoreactivity (in arbitrary units) in the sp5c. Mean ± SEM; one-way ANOVA; *n* = 3 rats per group; **P < 0.01, ***P < 0.001. **J** Quantification of FKN immunoreactivity (in arbitrary units) in the sp5c. Mean ± SEM; one-way ANOVA; *n* = 3 rats per group; **P < 0.01, ***P < 0.001

**Fig. 4:**
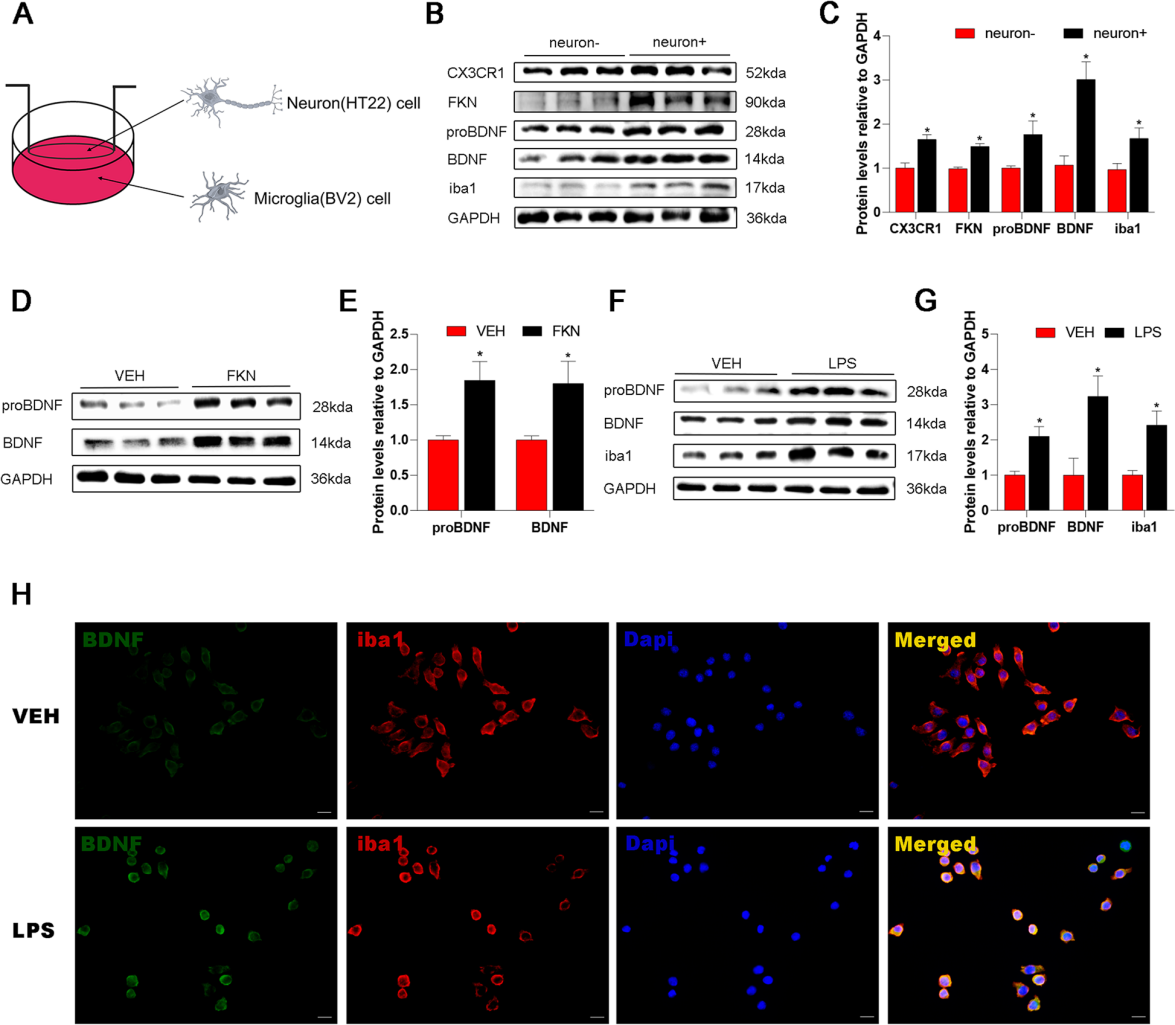
Neurons interact with microglia via the FKN/CX3CR1 axis in HT22-BV2 cocultures and enhance the release of BDNF by BV2 microglia. **A** Diagram of the HT22-BV2 coculture experiment. **B** Representative western blot images showing CX3CR1/FKN/proBDNF/BDNF/iba1 protein produced by BV2 cells in the presence of HT22 cells. **C** Quantification of CX3CR1/FKN/proBDNF/BDNF/iba1 protein levels in neuron- and neuron + cultures. Mean ± SEM; unpaired t test versus neuron- group; *n* = 3 rats per group; *P < 0.05. **D** Western blot images showing proBDNF/BDNF/iba1 protein produced by BV2 cells in response to FKN stimulation. **E** Quantification of proBDNF and BDNF protein levels in the VEH group and FKN group. Mean ± SEM; unpaired t test versus VEH group; *n* = 3 rats per group; *P < 0.05. **F** Representative western blot films showing proBDNF, BDNF, and iba1 protein produced by BV2 cells in the VEH group and the LPS group. **G** Western blot analysis showed that the proBDNF, BDNF, and iba1 protein levels in the LPS group were upregulated compared to those in the VEH group. Mean ± SEM; unpaired t test versus VEH group; *n* = 3 per group; *P < 0.05. **H** Representative immunofluorescence staining of BDNF (green) and iba1 (red) in the VEH and LPS groups. Scale bar = 20 μm. VEH, vehicle

**Fig. 5:**
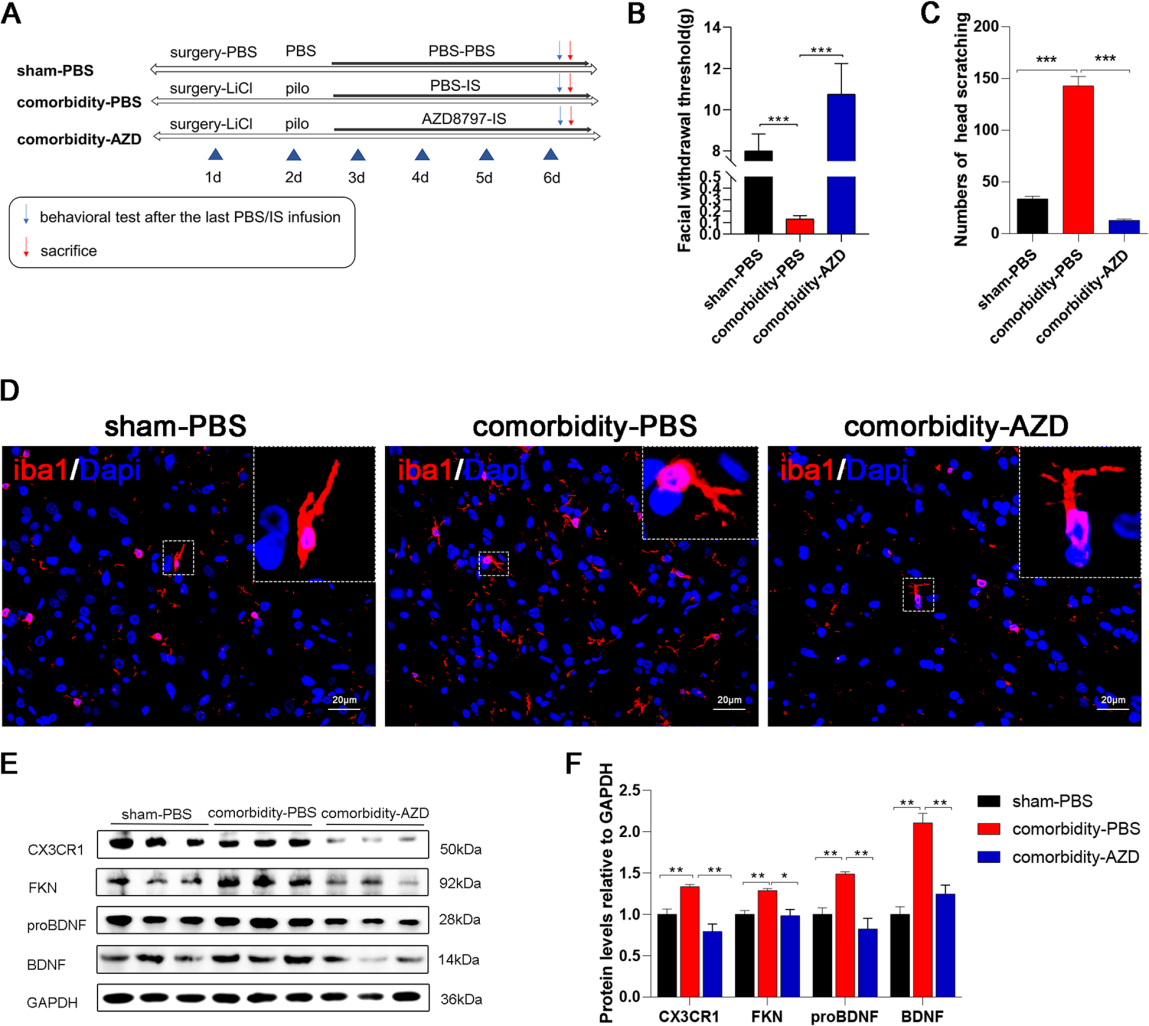
AZD8797 treatment attenuates SE-induced increases in nociceptive behaviour, microglial activation, and protein changes. **A** Flow chart of the experimental groups: sham-PBS, comorbidity-PBS, and comorbidity-AZD. **B** Mechanical threshold in the comorbidity-PBS group was decreased compared to that in the sham-PBS group and was reversed in the comorbidity-AZD group. Mean ± SEM; one-way ANOVA; *n* = 6 rats per group; ***P < 0.001. **C** Head-scratching counts were increased in the comorbidity-PBS group compared to the sham-PBS group, and were restored after AZD8797 treatment. Mean ± SEM; one-way ANOVA; *n* = 6 rats per group; ***P < 0.001. **D** Immunofluorescence staining of iba1 showing that AZD8797 reversed epileptic microglial activation and hyperplasia. Scale bar = 20 μm. The white box was enlarged. **E** Representative western blot images showing CX3CR1/FKN/proBDNF/BDNF/iba1 protein expression in the sp5c of the different groups. **F** Quantification of proBDNF and BDNF protein levels in the sham-PBS group, comorbidity-PBS group, and comorbidity-AZD group. Mean ± SEM; one-way ANOVA; *n* = 3 rats per group; *P < 0.05, **P < 0.01

1. **Evaluation of the rat models**
  After all groups were administered drugs or underwent surgery, we assessed the effectiveness of modelling and mortality. A total of 32 rats were treated with pilocarpine throughout the experiment. These were the SE group, the comorbidity group, the comorbidity-PBS group, and the comorbidity-AZD group. Twenty-six rats were successfully modelled, and the success rate was approximately 81%. In total, two rats died due to epileptic seizures (1 rat died of prolonged seizures caused by failure to stop them in time, and one died of spontaneous seizures that occurred after the last injection of IS). Four rats had seizures that did not reach grade 4/5 according to the modified Racine scale. In the remaining six groups of model rats, three rats died of surgical wound infection after modelling; seven rats were eliminated based on the nociceptive behaviour relevant to migraine.
2. **SE increased the nociceptive behaviour in migraine model rats**
  To investigate the impact of seizures on migraine, we established a comorbidity model of migraine and epilepsy as previously described (Fig. 1A) [25]. Consistent with our previous results, the migraine group had a lower Von-Frey filament force (1.12 ± 0.19) and a significantly higher number of head scratches (83.01 ± 1.92) than the sham group (7.98 ± 0.82; 33.82 ± 2.39; Fig. 1B-C). The results indicate that the migraine model was successfully stablished. Moreover, SE exacerbated the increased nociceptive behaviour, and there was a marked decrease in the periorbital mechanical threshold (0.11 ± 0.03) and a 1.5-fold increase in head-scratching counts compared to those in the migraine group (Fig. 1B-C). Although the pain-related behaviour of the SE group were also reduced, they were not significantly different from those of the sham group. To further verify these effects, the protein expression of cfos, an immediate early gene product that is often used as a marker of neuronal activation, was measured. The mean density of cfos in the sp5c was increased in the migraine group (83.91 ± 0.35) and further increased in the comorbidity group (97.43 ± 0.56) compared to the sham group (80.20 ± 0.35; Fig. 1D-E). Western blot analysis also demonstrated that cfos protein levels were increased in the comorbidity group (2.83 ± 0.09) compared to the migraine group (1.82 ± 0.04) and the sham group (1.01 ± 0.02; Fig. 1F-G). These results indicated that the activation of the trigeminal pathway in migraine model rats was exacerbated after seizures.
3. **The number and activation state of microglia was increased in the cortex/thalamus/sp5c after seizures**
  Gliosis is considered a molecular hallmark of status epilepticus [35]. Previous studies have demonstrated that microglial activation occurs in both epilepsy and migraine [17, 36]. To explore microglial changes after epileptic seizures and whether these changes were associated with the occurrence of migraine, immunohistochemical analysis of iba1, a marker of microglia, was performed to assess microgliosis. IHC analysis showed that the number of microglia in the SE group selectively increased along the trigeminovascular pathway (Fig. 2A-C), which is related to the pathogenesis of migraine. Microgliosis was present in the temporal lobe cortex (a 2.8-fold increase), paraventricular thalamic nucleus (PV, a 3.2-fold increase), laterodorsal thalamic nucleus, ventrolateral part (LDVL, a 1.4-fold increase), ventromedial thalamic nucleus (VM, a 2.2-fold increase), and sp5c (a 1.3-fold increase). Moreover, Sholl analysis of microglia confirmed that the complexity and activation state of microglia were increased after seizures. The SE group had larger cell bodies (6.58 ± 0.78, *P* < 0.01), more synapses (55.43 ± 5.6, *P* < 0.01), and shorter branches (15.82 ± 1.51, *P* < 0.01) than the sham group (Fig. 2D-E). To further verify protein expression in microglia in the cortex/thalamus/sp5c, we also measured the levels of iba1 protein (Fig. 2F-G). The results further confirmed the previous results. The protein levels of iba1 in the SE group were approximately 1.2-fold higher than those in the sham group (Fig. 2F-G).
4. **Epileptic seizures increased FKN/CX3CR1 expression in the cortex/thalamus/sp5c of migraine model rats**
  FKN/CX3CR1 signalling is a vital pathway that regulates microglial migration and function in the brain [37]. To determine whether the FKN/CX3CR1 axis is involved in the pathological mechanism of migraine after epilepsy, we measured the protein levels of FKN and CX3CR1 in the temporal cortex, thalamus, and sp5c of SE model rats four days after seizures (Fig. 3A). Compared to those in the sham group, CX3CR1 protein levels were significantly increased to 117% in the temporal cortex, 128% in the thalamus, and 181% in sp5c (Fig. 3B). While FKN expression increased to 108% in the cortex, 180% in the thalamus, and 110% in the sp5c (Fig. 3C). Then, we assessed FKN/CX3CR1 expression in the migraine and comorbidity groups. Compared with that in the sham group, the expression of CX3CR1/FKN in the sp5c was increased in the migraine group and further increased in the comorbidity group (Fig. 3D-E; F_CX3CR1_ = 21.29, *P* < 0.001; F_FKN_ = 24.12, *P* < 0.001). These results suggest that the FKN/CX3CR1 axis may be linked to the pathogenesis of migraine and that epileptic seizures may exacerbate migraine by promoting the expression of FKN/CX3CR1 axis. Furthermore, immunofluorescence staining was conducted to examine the FKN/CX3CR1 expression in the sp5c. With iba1 immunoreactivity serving as a marker for microglia, a marked visual overlap between iba1 and CX3CR1 immunoreactivities was observed in sp5c sections (Fig. 3F). Compared to those in the sham group, CX3CR1 and iba1 immunoactivities were considerably higher in the migraine group and even higher in the comorbidity group. (Fig. 3H-I; F_CX3CR1 intensity_ = 12.52, *P* < 0.01; F_iba1_ intensity = 8.15, *P* < 0.01). Correspondingly, FKN was mainly expressed in the cytoplasm of neurons and largely colocalized with Neun, a neuronal marker (Fig. 3G). Immunostaining analysis showed that FKN expression was increased a nearly 2.0-fold in the migraine group and approximately 2.2-fold in the comorbidity group compared to the sham group (Fig. 3J).
5. **Neurons activated microglia by releasing FKN and increased BDNF synthesis in HT22-BV2 coculture cells**
  To determine the relationship between microglial activation and the FKN/CX3CR1 axis, we indirectly cocultured BV2 microglial cells in transwell plates with HT22 neurons. In the transwell coculture system, neurons and microglial cells shared the same medium, but no direct cell–cell interactions were possible (Fig. 4A). After 24 h of coculture, the protein levels of the underlying BV2 cells were measured by western blotting. Compared with BV2 cells that were cultured alone, BV2 microglial cells in the coculture system had a nearly 3.0-fold increase in BDNF, while the levels of CX3CR1/FKN/proBDNF/iba1 increased by approximately 1.5-fold (Fig. 4B-C). To further confirm whether the upregulation in proBDNF/BDNF levels upregulation was caused by FKN released by neurons in the upper layer, we stimulated BV2 microglia with 0.1 mg/ml FKN for 24 h and examined BV2 morphologic changes. The protein levels of proBDNF increased by 1.2-fold, and BDNF increased by 1.8-fold after FKN treatment compared to those in the vehicle (VEH) group (Fig. 4D-E). These results suggest that microglia produce and release BDNF due to the interaction between neurons and microglia, and the FKN/CX3CR1 axis most likely mediates this interaction. In addition, to investigate whether the change in proBDNF/BDNF levels was related to microglial activation, we stimulated microglia with LPS, a recognized activator of microglia. LPS group cells were treated with 0.1 mg/ml LPS for 90 min, and morphological changes were observed and showed activated BV2 cells, while control BV2 cells were treated with a matching vehicle (VEH). Compared with those of resting microglia, the levels of proBDNF and BDNF secreted by activated microglia was significantly increased by 2.0-fold and 3.0-fold, respectively (Fig. 4F-G). Iba1 levels also showed a 2.5-fold increase compared to those in the VEH group. Moreover, immunofluorescence staining confirmed these results and showed a high level of colocalization between iba1 (red) and BDNF (green) (Fig. 4H).
6. **AZD8797 treatment reduced nociceptive behaviour and proBDNF/BDNF/iba1 expression in migraine model rats after SE**
  AZD8797 is one of the most commonly used antagonists of CX3CR1[32]. To further explore the effects of the FKN/CX3CR1 axis on nociceptive behaviour after an epileptic seizure, AZD8797 was injected into SE model rats once per day for four days before dural administration of IS to inhibit the FKN/CX3CR1 axis (Fig. 5A). The comorbidity-AZD group (10.82 ± 1.5) had significantly greater improvements in pain threshold than the comorbidity-PBS group (0.13 ± 0.03). There were no differences in the sham-PBS group (8.02 ± 0.82, Fig. 5B). Moreover, the number of head scratchings in the comorbidity-AZD group (12.81 ± 1.3) was decreased compared to that in the comorbidity-PBS group (114.24 ± 29.4, Fig. 5C). These results indicate that AZD8797 reversed the decline in nociceptive behaviour in comorbid rats. To further verify the role of FKN in the regulation of microglia, the expression levels of iba1 in the sp5c were examined by immunofluorescence staining. The results showed that AZD8797 reduced the number of iba1-positive microglia and microglial activation in rats with migraine after seizures (Fig. 5D, *P* < 0.01). We next explored the protein expression of CX3CR1/FKN/proBDNF/BDNF to examine the effects of AZD8797 on migraine model rats after seizures at the molecular level. The expression of FKN/CX3CR1 axis factors decreased by 1/3 in the comorbidity-AZD group compared with the comorbidity-PBS group (Fig. 5E-F, *P* < 0.01), indicating that AZD8797 could block the FKN/CX3CR1 axis. Additionally, the expression of proBDNF and BDNF decreased by 45% and 41%, respectively, after AZD8797 treatment (Fig. 5E-F). This finding further demonstrated that the FKN/CX3CR1 axis might act on migraine through the BDNF signalling pathway.
7. **FKN infusion increased nociceptive behaviour and proBDNF/BDNF/iba1 expression in migraine model rats**

**Fig. 6:**
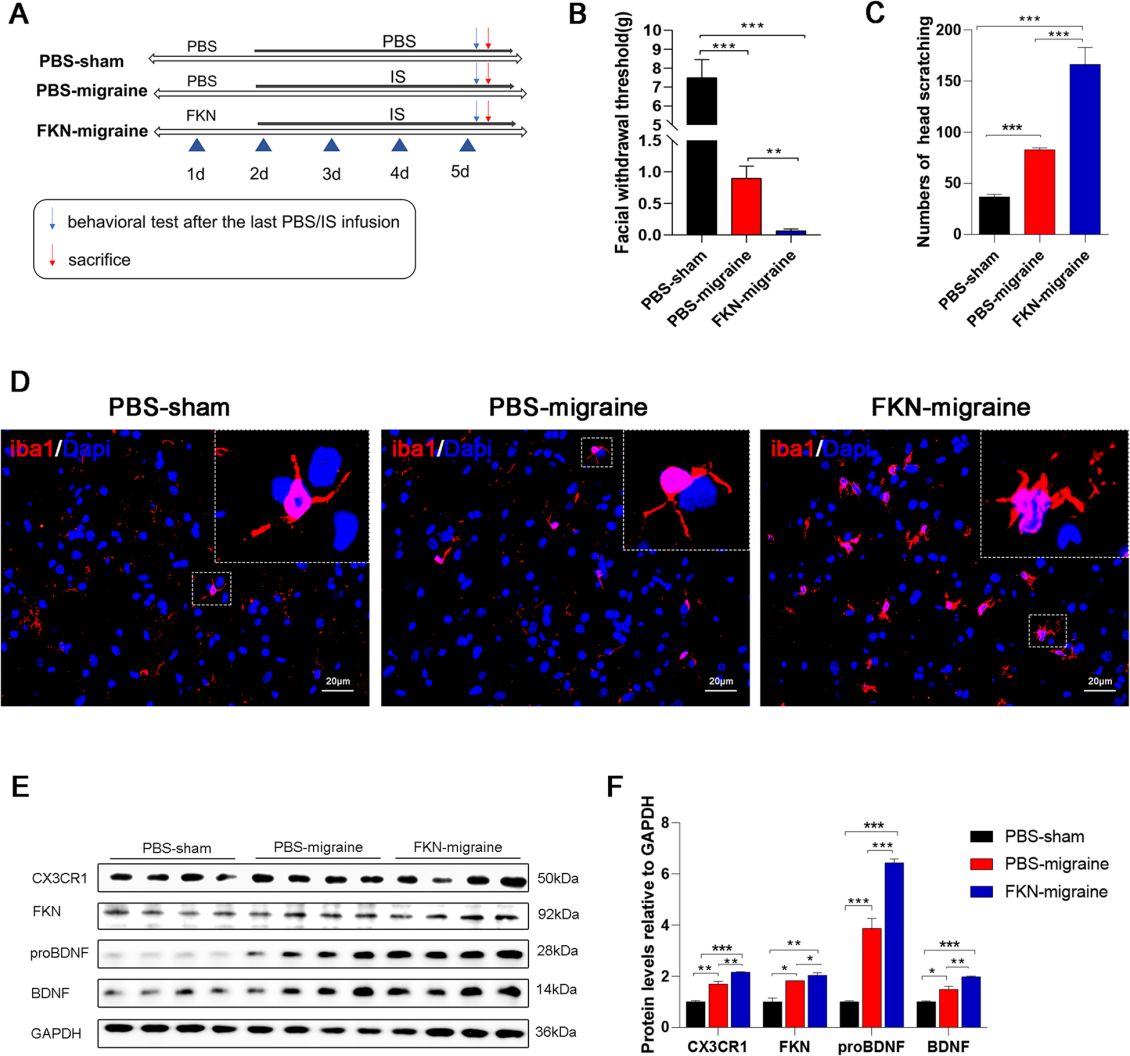
FKN infusion exacerbated the SE-induced increase in nociceptive behaviour, microglial activation, and relative protein upregulation. **A** Flow chart showing PBS-sham, PBS-migraine, and FKN-migraine experimental groups. **B** The mechanical threshold in the PBS-migraine group was decreased compared to that in the PBS-sham group and further decreased in the FKN-migraine group. Mean ± SEM; one-way ANOVA; *n* = 6 rats per group; **P < 0.01, ***P < 0.001. **C** Head-scratching counts were increased in the PBS-migraine group, compared to the PBS-sham group and were further increased after FKN infusion. Mean ± SEM; one-way ANOVA; *n* = 6 rats per group; **P < 0.01, ***P < 0.001. **D** Immunofluorescence staining of iba1 shows that FKN exacerbated epileptic microglial activation and increased microglial numbers. Scale bar = 20 μm. The white box was enlarged. **E** Representative western blot films showing CX3CR1/FKN/proBDNF/BDNF/iba1 protein expression in the sp5c in different groups. **F** Quantification of CX3CR1/FKN/proBDNF/BDNF/iba1 protein levels in the PBS-sham group, PBS-migraine group, and FKN-migraine group. Mean ± SEM; one-way ANOVA; *n* = 3 rats per group; *P < 0.05, **P < 0.01, ***P < 0.001

To further investigate whether seizures facilitate migraines through the FKN/CX3CR1 axis, we injected FKN into the sp5c of migraine model rats using stereotactic brain injection, and the sham groups received the same amount of PBS. The experimental procedure is shown in Fig. 6A. Consistent with that of the comorbidity model rats, the mechanical pain threshold of the PBS-migraine group (0.94 ± 0.19) was decreased and was further decreased in the FKN-migraine group (0.12 ± 0.03) compared with the PBS-sham group (7.54 ± 0.96, Fig. 6B). Moreover, FKN infusion further increased the head-scratching counts of migraine model rats (*P* < 0.001; Fig. 6C). Iba1 immunofluorescence showed that FKN infusion activated sp5c microglia in migraine rats (*P* < 0.01; Fig. 6D). The protein levels of proBDNF/BDNF were also increased in the FKN-migraine group (6.44 ± 0.15; 1.87 ± 0.08) compared with the PBS-migraine group (3.92 ± 0.39; 1.64 ± 0.11, Fig. 6E-F). These results suggest that FKN can mimic the effect of epileptic seizures on migraine to some extent, suggesting that hyperexcited neurons after epileptic seizures may interact with microglia through the FKN/CX3CR1 axis to promote migraine.

## Discussion

Migraine comorbidity with epilepsy significantly increases disease burden. Although numerous studies have been published on the association of microglia with migraine and epilepsy, few studies have investigated whether microglial activation is one of the causes of migraine susceptibility after epileptic seizures. In line with this hypothesis, our study supports this idea by showing that neurons activate microglia in the cortex/thalamic/sp5c via the FKN/CX3CR1 axis, which leads to enhanced BDNF production in migraine model rats following an epileptic episode. Furthermore, we explored whether the FKN/CX3CR1 axis intervention regulated nociceptive behaviour in migraine model rats, further revealing its role in migraine. These findings contribute to advancing our understanding of the pathophysiological process and provide a potential preventative target for migraine after seizures.

In accordance with previous reports [38], we confirmed that epilepsy is accompanied by microglial activation and recruitment. The interaction between neurons and microglia is critical in this pathological process. In epilepsy, hyperexcitable neurons send signals to nearby microglia [2]. In response to stress, microglia transform from a surveillant to an activated phenotype, which involves changes in morphology and sustaining secondary neuroinflammation. Furthermore, we demonstrated that FKN/CX3CR1 plays an indispensable role in the communication between neurons and microglia. Consistent with our findings, numerous studies have revealed that increased fractalkine signalling plays a role in the epileptic brain. In patients with temporal lobe epilepsy (TLE), immunoreactivity and protein levels of FKN were increased in the temporal neocortex and hippocampus compared to nonepileptic autopsy controls [39]. In animal models of epilepsy, FKN levels increase 1–3 days after seizures [40]. However, FKN expression three days later is controversial. Yeo et al. showed that FKN decreases after three days [40], whereas Wu et al. believed that FKN continues to increase after 3 days but peaks at 72 h and remains relatively high for the next 60 days [41]. In our study, FKN levels on the fourth day after the seizure were higher than those in the control group (Fig. 3A-B). The difference may be due to the location of the measurement. With either analysis, it should be emphasized that FKN plays a role in the pathophysiological mechanism of epilepsy. Consistent with a previous study, the immunoreactivity and protein levels of CX3CR1 remained higher than those of the control at four days after SE (Fig. 3A-B). There is a monogamous association between FKN and CX3CR1 [21]. After epileptic seizures, the expression of FKN in neurons increased. By interacting with CX3CR1 expressed on the microglial membrane, FKN promoted microglial activation and recruitment in specific brain regions. This process may play a significant role in increasing migraine susceptibility following epilepsy. After the first seizure, activated microglia make the epileptic brain hypersensitive and are more likely to cause migraine headaches in an externally stimulated or hyperimmune environment than in a healthy brain. Although there have been few studies on the FKN/CX3CR1 axis in migraine, published research suggests that CX3CR1 may be involved in the occurrence of migraine [42]. Consistent with our results, another group postulated that microglial activation mediated by CX3CR1 represents a distress signal that leads to headache. This may be one of the explanations for the predisposition of epileptic patients to migraines. To our knowledge, we are the first group to link FKN signalling to postseizure migraine. Further studies are still needed to explore the association and specific mechanism between them.

Even more unexpected was that the activation and proliferation of microglia after epileptic seizures are region-specific, and these cells were mainly concentrated in the cortex, and the thalamic nucleus (Fig. 2A), which are closely related to pain attacks. This finding further demonstrates that microglial activation plays a role in the association between epilepsy and migraine. Consistent with our results, a recent study reported that mean iba1 labelling in all brain regions was significantly increased in patients with epilepsy. When epilepsy causes sudden and unexpected death, the medial thalamic nucleus and superior temporal gyrus alternatively increase [43]. Furthermore, another study that used translocator protein-targeted molecular imaging showed microglial activation in the hippocampus, thalamus, and piriform cortex in LiCl-pilo-induced SE model rats [44]. Compared to these studies, our study is more specific in describing the site of microglial activation and proliferation in the thalamus after epileptic seizures. The specific mechanism of the above nuclei (PV, LDVL, and VM) in migraine and epilepsy is now still under investigation in our laboratory. Previous studies have demonstrated that the thalamus acts as the main gate, filtering and processing sensory inputs from sensory and associative neocortical projections [20]. This result means that the thalamus plays a role in seizures and migraines. During a migraine or seizure attack, major suppression of depolarization occurs in the cortex. The thalamus monitors changes in cortical signals and sends them to peripheral sensory areas such as the trigeminal nerve. Microglia monitor changes in the thalamic cortical network and make adaptive adjustments, such as releasing inflammatory factors, which in turn worsen seizures and migraines. Notably, we also focused on changes in microglia in the sp5c after epileptic seizures. In our study, the microglia in cortical and thalamic nuclei showed apparent accumulation and proliferation, while the sp5c only had an increase in number without microglial recruitment. And perhaps migraines are triggered in epilepsy patients only when the number of activated microglia in the sp5c reaches a specific threshold. This might explain why not all epilepsy patients suffer from migraines. Further studies are necessary to substantiate this perspective and further investigate the connection between the cortex, thalamus, and sp5c.

Our previous studies have shown that BDNF is involved in comorbidities of epilepsy and migraine [26]. This study further shows that activated microglia facilitate migraine by synthesizing and releasing BDNF after epileptic seizures. Current opinion tends to consider BDNF to be a pain modulator [45]. During a headache, the afferent input from the meninges primes the dural nociceptive system using the central release of BDNF [46]. Then BDNF further increases excitability and signalling of pain pathways through altered glutamatergic and GABAergic/glycinergic signals [47]. When combined with our findings, it is reasonable to hypothesize that microglia- produced BDNF may play a vital role in the development of postepileptic migraine. We also demonstrated that BDNF expression was regulated by the FKN/CX3CR1 axis in migraine model rats. In accordance with our results, Tan et al. showed that BDNF expression was decreased in CX3CR1-knockout mice [48]. Wang et al. also showed that microinjection of FKN into the hippocampus rescued hippocampal-dependent memory in BDNF Val66Met mutant mice [49]. Another study demonstrated that blocking CX3CR1 decreased the BDNF level in rat brain tissue [50]. However, challenges remain in translating results from animal experiments into human therapies because some studies have shown that there are differences in other downstream signalling mechanisms of CX3CR1 in humans and rodents [51]. Further studies are needed to verify whether the FKN/CX3CR1 axis is a feasible therapeutic target in human patients.

The present study provides evidence that modulating microglial activation can affect epileptic seizure-induced migraine-relevant phenotypes. These results deepen the understanding of the role of microglia in epilepsy, fill the gap in the research of microglia in the comorbidity of epilepsy and migraine, and provide a new approach for managing migraines after epilepsy. However, this study has limitations that must be acknowledged. First, microglia may play dual roles role in the pathogenesis of epilepsy. Wu et al. discovered that microglial depletion exacerbates the severity of acute and chronic seizures in Cx3cr1-KO mice [52]. This finding suggests that complete ablation of microglia can also impair the development and function of the central nervous system. It also suggests that using large doses of FKN/CX3CR1 inhibitors in individuals with epilepsy and migraines may not alleviate migraines and worsen seizures. Therefore, it is still necessary to further explore the concentration and administration of FKN/CX3CR1 inhibitors in clinical and animal experiments. Second, only male rats were included to analyse neural mechanisms. Sexual dimorphism in FKN and BDNF in the central nervous system has been suggested in some studies [53, 54]. It is now generally accepted that migraines are more common in women than in men. The role and neural function of FKN in female migraine still need to be further investigated.

## Conclusions

In summary, through a rat model of comorbid migraine and epilepsy, we explored the characteristics and mechanism of microglial activation after seizures and their effects on migraine. These results indicate that SE may facilitate migraine through BDNF release induced by microglial activation in the cortex/thalamus/sp5c mediated by the FKN/CX3CR1 axis. Future studies are needed to illustrate the feasibility of targeting the FKN/CX3CR1 axis in migraine patients, especially in female patients.

## Supplementary Information

**Additional file1: Fig. S1.** Schematic representation of the FKN injection site.

## Data Availability

The datasets used and analyzed in the present study are available from the corresponding author on reasonable request.

## Abbreviations

BDNF: Brain-derived neurotrophic factor
BSA: Bovine serum albumin
CNS: Central nervous system
CSD: Cortical spreading depression
FKN: Fractalkine
fMRI: Functional magnetic resonance imaging
ICC: Immunocytochemistry
IF: Immunocytochemistry
IHC: Immunohistochemistry
IS: Inflammatory soup
LDVL: Laterodorsal thalamic nucleus, ventrolateral part
LiCl: Lithium chloride
LPS: Lipopolysaccharide
PBS: Phosphate buffer saline
PMSF: Phenylmethylsulfonyl fluoride
proBDNF: Precursor molecule Brain-derived neurotrophic factor
PV: Paraventricular thalamic nucleus
PVDF: Polyvinylidene difluoride
SD: Sprague-Dawley
SE: Status epilepticus
sp5c: Spinal trigeminal nucleus caudalis
SPF: Specific pathogen-free
TLE: Temporal lobe epilepsy
VEH: Vehicle
VM: Ventromedial thalamic nucleus
WB: Western blotting
