# Supplementary figures and images for: FKN/CX3CR1 axis facilitates migraine-Like behaviour by activating thalamic-cortical network microglia in status epilepticus model rats
Source: J Headache Pain. 2022 Apr 5;23(1):42. doi: 10.1186/s10194-022-01416-w (PMC8981829; doi:10.1186/s10194-022-01416-w)

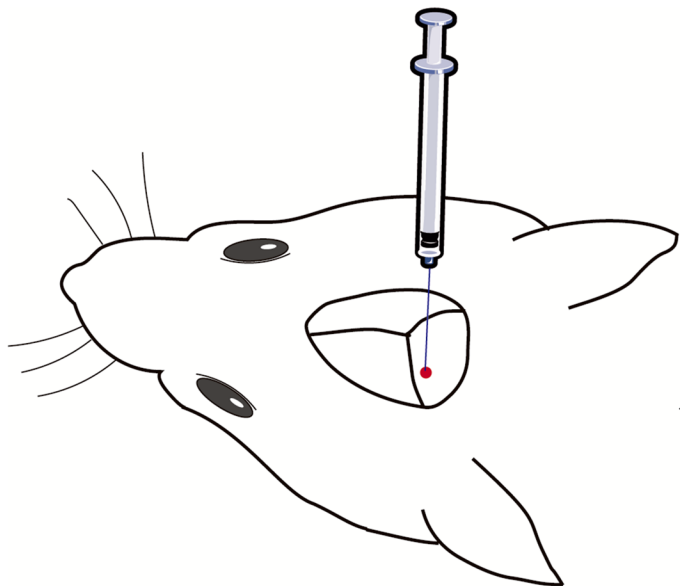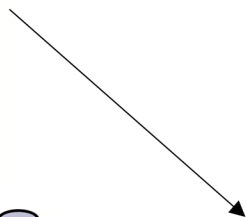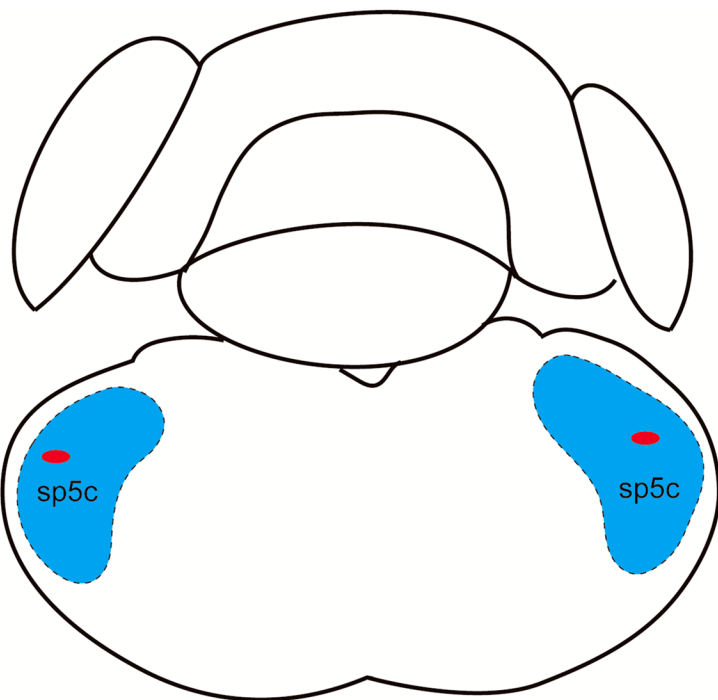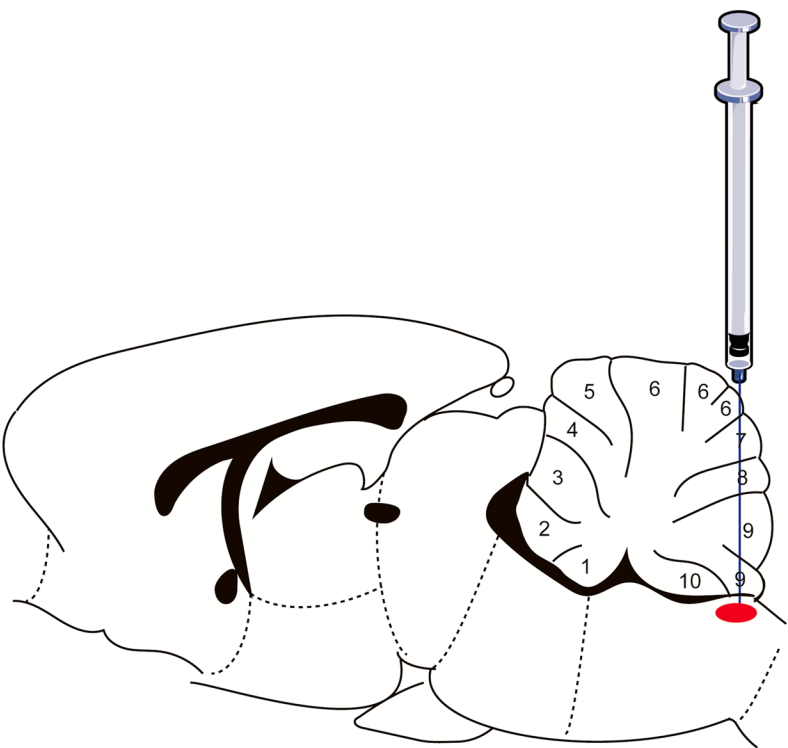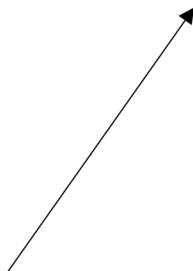

Supplement: Supplementary file 1 — Additional file1: Fig. S1. Schematic representation of the FKN injection site. [file 10194_2022_1416_MOESM1_ESM.pdf]
